# Multifunctional Nanomaterials: Design, Synthesis and Application Properties

**DOI:** 10.3390/molecules22020243

**Published:** 2017-02-07

**Authors:** Marisa Martinelli, Miriam Cristina Strumia

**Affiliations:** Departamento de Química Orgánica (IPQA, CONICET-UNC), Facultad de Ciencias Químicas, Universidad Nacional de Córdoba, Córdoba X5000HUA, Argentina; mmartinelli@fcq.unc.edu.ar

**Keywords:** dendrons, dendronization, dendronized materials, hybrid materials

## Abstract

The immense scope of variation in dendritic molecules (hyper-branching, nano-sized, hydrophobicity/hydrophilicity, rigidity/flexibility balance, etc.) and their versatile functionalization, with the possibility of multivalent binding, permit the design of highly improved, novel materials. Dendritic-based materials are therefore viable alternatives to conventional polymers. The overall aim of this work is to show the advantages of dendronization processes by presenting the synthesis and characterization of three different dendronized systems: (I) microbeads of functionalized chitosan; (II) nanostructuration of polypropylene surfaces; and (III) smart dendritic nanogels. The particular properties yielded by these systems could only be achieved thanks to the dendronization process.

## 1. Introduction

Nature is the source of inspiration for the development of new materials that meet the increasing demands of complex and highly specific systems. In this context, scientists are now focused on developing highly branched and functionalized structures. The final properties and potential applications of these types of materials are determined by their architecture and functionalities.

Historically, polymer science has been mainly based on linear, crosslinked, or branched networks, with no strict control over the architecture. Dendritic polymers, however, have found a superior place in polymer technology. Dendritic molecules are characterized by dense and less dense zones, depending upon the rigidity and/or conformational mobility of their scaffold. Thus, these structures can form cavities to accommodate solvents and can act as selective host compounds for guest substances. In contrast to conventional polymers, high local concentrations of certain structural elements and functionalities can be obtained, making them particularly suitable for applications in the biomedical sector [1]. Dendritic-based materials are therefore potential alternatives to conventional polymers [2].

Nevertheless, the synthesis of dendrimers has certain drawbacks related to the high production costs and the need for the skills of an organic chemist. Traditional syntheses involve repetitive stepwise growth and deprotection/activation stages with careful purification procedures. Dendronization overcomes these disadvantages [3] and consists of the covalent or physical interaction of dendrons with non-dendritic substrates (organic, inorganic, or hybrid) to create very well-defined, stable molecular level nanostructures with dendritic effects [4].

**Figure 1:**
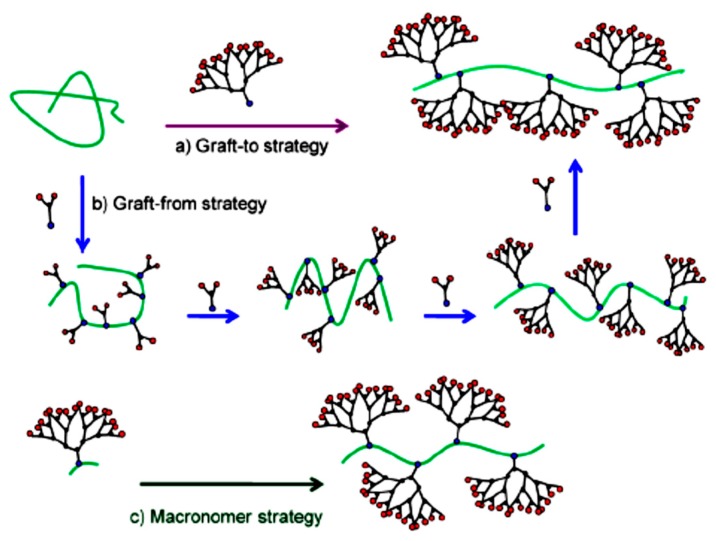
Schematic illustration of the three general synthetic strategies for dendronized polymers: (**a**) graft-to; (**b**) graft-from; (**c**) macromonomer route. Reproduced from Julieta I. Paez et al. [3].

Synthetic strategies of dendronized polymers can be divided into four main categories: the coupling of suitably functionalized fragments; the growth of dendritic molecules from the linear segments; the growth of a linear chain from the reactive groups of dendrimers; and the polymerization of macromonomers containing dendritic and linear blocks (dendron with polymerizable group) [4,5] (Figure 1).

The choice of dendrons plays a very important role in the management of the structure/property relationship of the substrate in which the dendronization process is applied. Certain aspects of the behavior of dendrons are dominated by structural and geometric features, and can be divided into three groups: (a) the chemical structure of the dendron; (b) the generation or size of the dendron; and (c) the degree of dendronization.

**Figure 2:**
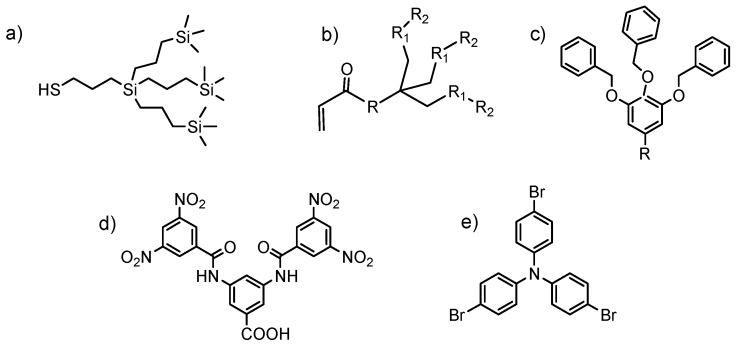
Dendrons used as building blocks, spacer arms, or functionalizing agents: (**a**) carbosilane dendrons; (**b**) Newkome-type dendrons; (**c**) Fréchet-type dendrons; (**d**) Kakimoto-type dendrons; (**e**) Triarylamine dendrons.

**Figure 3:**
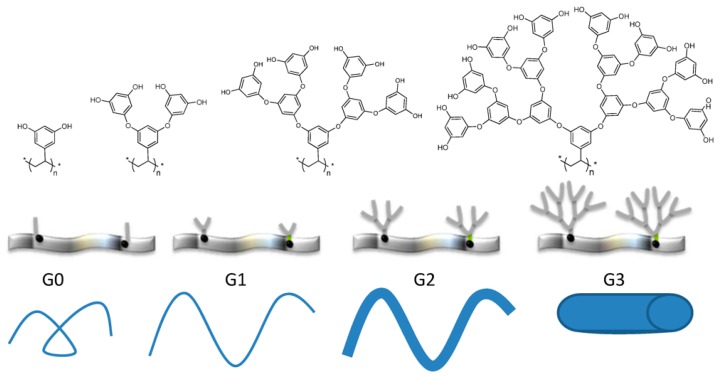
Scheme showing the variation of the structural flexibility of a dendronized polymer as a function of dendron generation (G0 to G3). Reproduced from Julieta I. Paez et al. [3].

**Figure 4:**
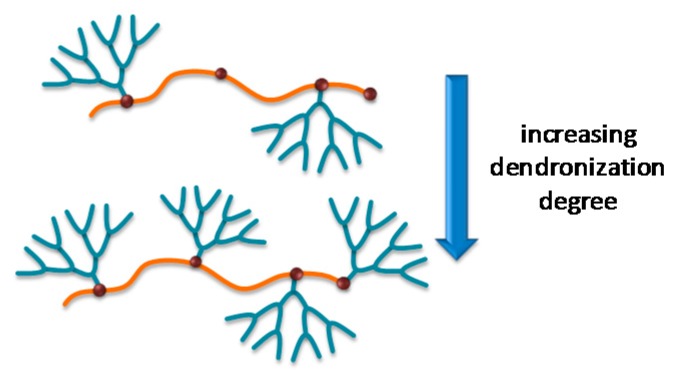
Schematic representation of the dendronization degree.

(a) Chemical structure of the dendron: this relates to the structural and chemical characteristics of the dendrons such as peripheral functional groups, focal point, junctions, and generation. The presence of different functional groups introduces specific properties into the material such as pH sensibility, hydrophilicity, hydrophobicity, ionic nature, and the possibility of using surface groups to couple dyes, metals, ligands, and other functionalities which are easily accessible to the environment. Figure 2 shows dendrons with a wide variability of the functional groups in their structures.
(b) Dendron generation or size: surface rigidity can be manipulated through dendron generation. For example, when the substrate is a linear polymer, the dendronized polymer can vary from a random coil conformation to rigid when the generation is increased [6] (Figure 3).
(c) The degree of dendronization, defined as the number of dendrons per unit of volume or mass (Figure 4) [7], affects the properties conferred on the surface, known as the dendritic effect. A large number of surface groups on the periphery is one of the most important structural characteristics of the dendronization process.

Several published papers have demonstrated the advantages of dendronization effects, and many authors have made use of them to change or introduce new properties in the starting materials [8,9,10,11,12].

Our research group is carrying out pioneering work in the development of dendronized materials for different applications. A family of dendrons has been synthesized (Newkome-type and Kakimoto-type) and has been used to modify different surfaces with the aim of manipulating the structure/property ratio in the search of specific properties for the final materials [13,14,15,16,17].

In previous studies reported by our group, we highlight the importance of dendronization as a tool for designing tailor-made polymeric materials with specific properties. The overall aim of this paper is to show the advantages of the dendronization process by presenting the synthesis and characterization of three different dendronized systems: (I) microbeads of functionalized chitosan; (II) nanostructuration of polypropylene surfaces and (III) smart dendritic nanogels.

In all three systems, the same dendron, tris[(tertbutoxycarbonyl) ethyl]aminomethane, so-called Behera’s amine, or derivatives were used to modify the substrates, but different methodologies were applied to introduce them onto the surfaces. We therefore maintained the chemical structure and generation of the dendron but modified the spatial distribution of the dendrons for each system. We used dendrons as tailored building blocks for the application of different dendronization methodologies in order to gain a deeper understanding of the control and tunability underpinning the dendronization process. Furthermore, we highlight the potential applications of dendronized materials. In summary, the importance of this study lies in showing that through the synthesis of three different systems by applying different dendronization methodologies, the properties observed in the final materials are a direct product of the selected dendronization process that is carefully applied in each of the systems.

## 2. Different Dendronized Systems

### 2.1. Microbeads of Functionalized Chitosan (System I)

**Figure 5:**
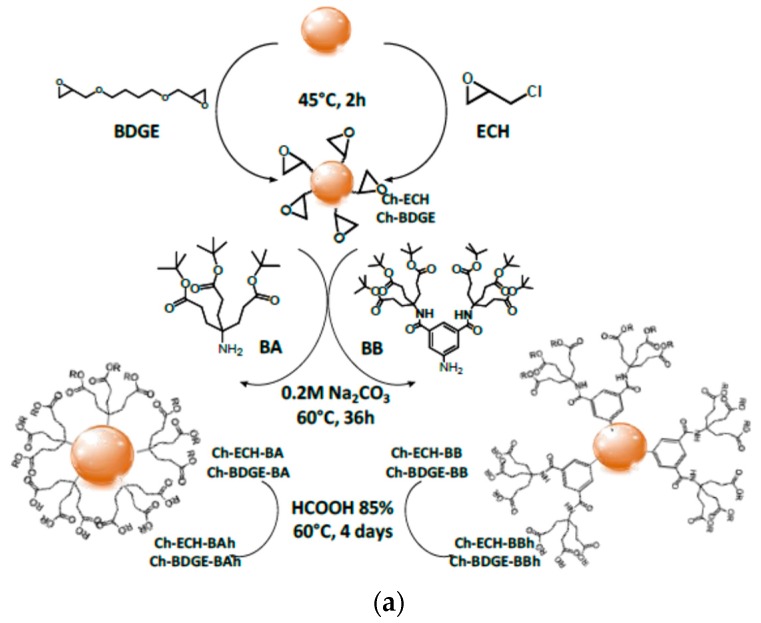

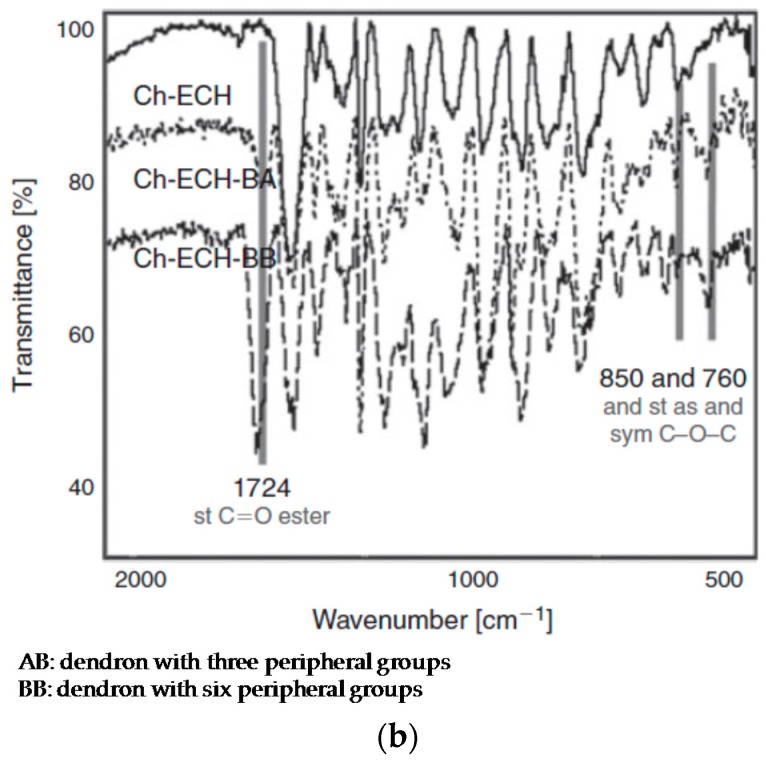
Synthetic route of dendronized microbeads (**a**); FT-IR spectra of dendronized microbeads (**b**). Reproduced from Ana Agustina Aldana et al. [14].

Microbeads of dendronized chitosan were synthesized and characterized in order to evaluate the dendritic effect [14]. The microbeads were dendronized on their surfaces with *tris*- and hexa-functionalized dendrons (AB and BB: Behera’s amine and derivatives) capable of chelating copper. Their application properties for use as catalysts were then evaluated. The coacervation/precipitation method [18] was used to prepare the microbeads (diameters between 0.8 and 1.2 mm) and the surface functional groups of chitosan were activated with epoxy groups (epichlorohydrin, ECH, or butylene diglycidyl ether, BDGE) for further linkage to the dendron, shown in Figure 5a. The microbeads were characterized by infrared spectroscopy, scanning electron microscopy, thermogravimetry, swelling capacity analysis, and atomic absorption spectroscopy. Figure 5b shows the FT-IR spectra of microbeads as an example. The properties of the material (hydrophilic–hydrophobic balance) and its ability to chelate copper were controlled by the size of the dendron and the number and types of functional groups at the periphery (ester or acid). The hydrolysis of the ester groups on the periphery depends on the spacer agent (ECH or BDGE) and on the number of groups on the periphery of the dendron (three or six). A combination of the hydrophilicity imparted by the linker agent and the steric hindrance could explain the highest percentage of hydrolysis observed for the microbeads modified with ECH and the dendron with six peripheral groups, respectively. The relative quantity of ester or acid groups on the periphery allowed control of the swelling degree at pH values (1.2 and 7.4) above and below the pKa of the amine groups of chitosan. The swelling degree of the dendronized microbeads modified with BDGE was driven by the free functional groups of the chitosan chain. On the contrary, the swelling behavior of the dendronized microbeads using ECH as the linker agent was governed by the groups of the periphery (ester or acid) of the dendron: at pH 1.2, the capacity to form hydrogen bonds between the acid groups of the periphery, due to their close proximity, hindered the entry of water into the network; at pH 7.4, the repulsive interaction of the carboxylate groups was the principal contribution.

**Figure 6:**
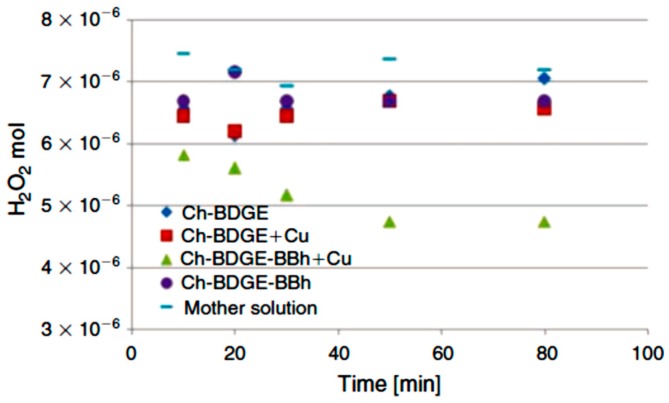
Reaction of hydrogen peroxide. Reproduced from Ana Agustina Aldana et al. [14].

The highest level of copper immobilization was obtained for chitosan dendronized with a lower degree of dendronization but using the dendrons with six periphery groups. A higher retention capacity of copper was attained with the microbeads dendronized with hexa-functionalized dendrons, even though the degree of dendronization was lower. This behavior could be ascribed to the characteristic cooperative effect of the dendronized system [19]. Additionally, an upper stability of the matrix–metal complex was observed for the dendronized microbeads. The catalytic activity of the dendronized beads was then assessed in the decomposition reaction of H_2_O_2_. The beads selected were those with higher copper binding but with a lower degree of dendronization, achieving a higher percentage of conversion than the reactions catalyzed by non-dendronized beads. Both metal coordination and catalytic activity were determined by the dendritic structure. As shown in Figure 6, after 80 min of the reaction, a low decomposition percentage (8%) was achieved for the reactions catalyzed by the control matrices, but 35% conversion was reached at 50 min with dendronized catalyst.

### 2.2. Nanostructuration of Polypropylene (PP) Surfaces (System II)

The nanostructuration of the PP surface was obtained through a two-stage process as shown in Figure 6. In the first step, titanium oxide nanoparticles (TiO_2_ NPs) were silanized in xylene solutions using (3-cloropropyl) trietoxisylane (CLE) with a 5/95 and 10/90 ratio of CLE/xylene. Then, the silanized NPs were modified by controlled radical polymerization (ATRP) using the aminotriester dendron (Behera’s amine) as the monomer (6 × 10^−5^ mol/mg NPs); the obtained products were designated NPs-g-PABA [20]. Chemical modifications of the NPs were corroborated by infrared spectroscopy, UV-Vis spectroscopy, transmission electron microscopy, thermogravimetric analysis, and small-angle X-Ray scattering. 

**Figure 7:**
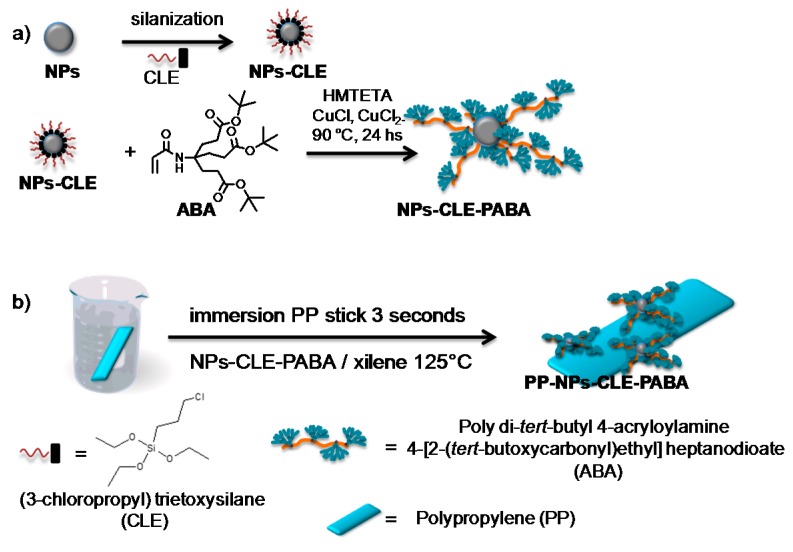
(**a**) TiO_2_ NPs functionalization with a dendronized polymer by controlled radical polymerization (ATRP); (**b**) superhydrophobic surfaces of polypropylene (PP) procedure.

**Figure 8:**
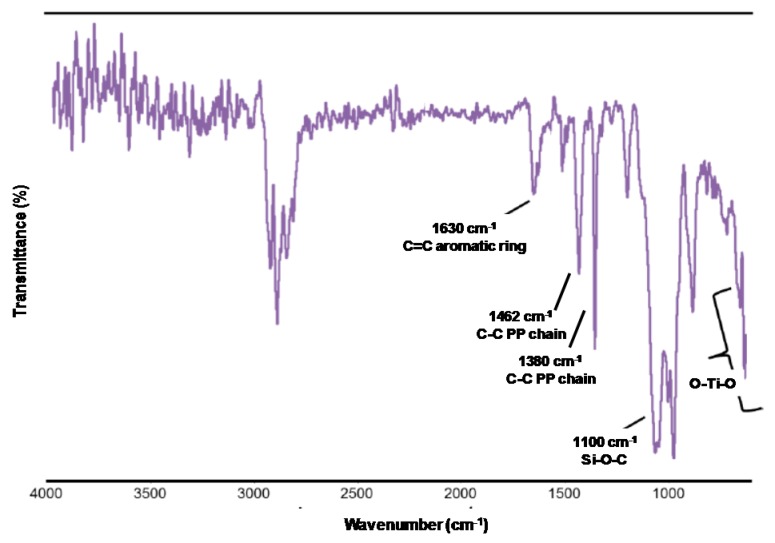
FT-IR spectrum of nanostructured PP with dendritic monomers.

In a second step, the TiO_2_ NPs were attached to the PP surfaces through a thermal adhesion process using a concentration of NPs-g-PABA in xylene of 10 mg/mL at 125 °C, obtaining the products designated PP-(NPs-g-PABA). The general scheme of the synthesis and experimental conditions is shown in Figure 7. The nanostructured PP surfaces were characterized by FTIR, scanning electron microscopy, profilometry, and contact angle (CA) and hysteresis contact angle (HCA) studies. Figure 8 shows the FT-IR spectrum of PP using NPs-g-PABA; the presence of the bands at 1720 cm^−1^ (-C=O of the dendron) corroborated the chemical modification of the PP surfaces. The dendronized NPs contained between 10% and 15% of organic coating.

Similar experiments were carried out using TiO_2_ NPs without chemical modification. These products (PP-NPs) were previously studied and reported [21], and were used for comparative analysis. In this study, the PP surfaces were modified using 1.5 and 15 mg/mL of NPs in xylene.

It is known [22,23,24] that the presence of a hydrophobic chemical component and a specific surface topology are required in order to obtain surfaces of a superhydrophobic nature. In our dendritic system, both requisites were fulfilled by the dendritic monomer: the presence of hydrophobic *tert*-butyl groups on the periphery of the dendron and a “branch-like” architecture on the surface of a nano-object such as TiO_2_ NPs. Furthermore, the superhydrophobic surfaces must have a CA larger than 150° and a HCA lower than 10°. Table 1 shows the comparative properties of all the systems studied, PP-NPs, and PP-(NPs-g-PABA).

The nanostructured surfaces of PP prepared using NPs without chemical modification showed superhydrophobic properties when a higher NPs/xylene concentration was used, while that in the dendritic system, in both silane/xylene ratios used, enabled us to obtain superhydrophobic surfaces. However, only using the highest silane/xylene ratio was it possible to also obtain self-cleaning properties (CA of 162° and hysteresis CA of 7.8°). The self-cleaning properties were not detected in the PP-NPs.

### 2.3. Smart Dendritic Nanogels (System III)

**Figure 9:**
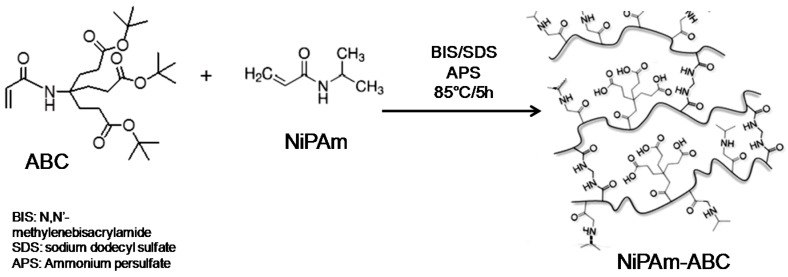
Copolymerization of *N*-isopropylacrylamide (NiPAm) with 4-acryloylamine-4-(carboxyethyl) heptanodioic acid (ABC). The synthesis of dendronized NGs.

This system presents the copolymerization of *N*-isopropylacrylamide (NiPAm) with aminotriacid dendron (Behera´s amine derivate) in order to yield smart nanogels (NGs) colloidally stable under physiological conditions [25], (Figure 9). 

**Figure 10:**
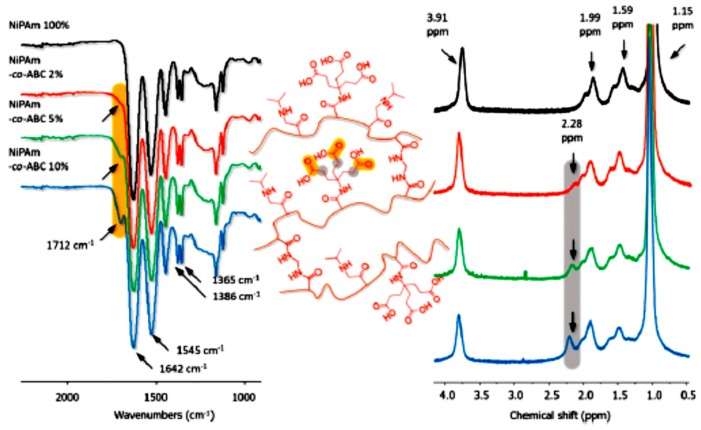
FT-IR (on the left) and ^1^H-NMR (on the right) spectra of NiPAm-based nanogel. Reproduced from G.N. Rimondino et al. [25].

The monomers incorporated into the polymer chain bestowed intelligent behavior on the nanogels against pH and temperature. The dendritic monomers with three carboxylic groups provided pH responsivity to a thermo-responsive NiPAm-based nanogel. The synthesis of the nanogels was carried out by precipitation polymerization at 88 °C, at temperatures above the lower critical solution temperature (LCST) of poly(NiPAm) [26,27]. Different dendritic monomer compositions in the NGs were evaluated (2%, 5%, and 10%) and the incorporation of monomers was confirmed by FT-IR and ^1^H-NMR spectroscopy, assigning the typical signals corresponding to NiPAm and the dendron, shown in Figure 10. The physico-chemical characterization and thermal and pH-triggered phase transitions were analyzed. The cloud point temperature (Tcp), which represents the phase transition temperature of the NGs, was determined by UV-Vis-based turbidity experiments and the diameters in aqueous solutions were measured by dynamic light scattering. NGs with low polydispersity were obtained, showing a complex phase transition regulated by temperature and pH.

The Tcp value was regulated by the dendritic moiety concentration and the intensity of the intermolecular hydrogen, which were strongest at low pH due to the inter- and intra-chain interactions between acid groups at the periphery of the dendron and NiPAm monomers, shown in Table 2.

A sharp, reversible pH-dependent phase change was obtained as a result of combining thermo-responsive NiPAm with dendritic ionizable monomers.

**Figure 11:**
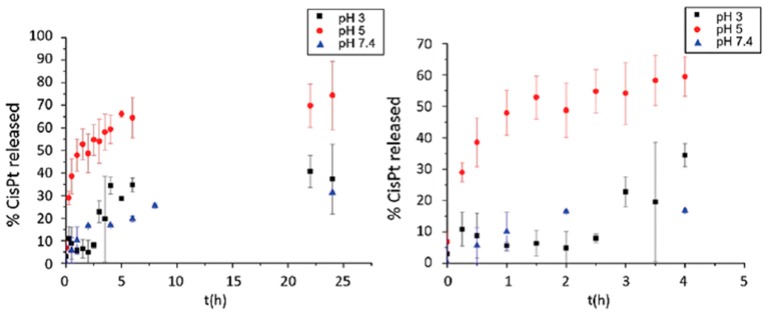
CisPt release from NiPAm-based nanogel (an enhanced image of the release plots is shown in the right panel). Adapted from G.N. Rimondino et al. [25].

In order to use this system as a drug delivery system, the loading and release of the antitumor drug Cisplatin and the in vitro cytotoxicity were studied with NiPAm-co-dendron 5% NGs; the results are shown in Figure 11. The hydrophilic/hydrophobic balance of the dendron determined the drug release kinetics, since the release was pH-dependent in vitro. The drug release was higher at a pH approaching the p*K*_a_ of the acid monomers (pH = 5) [28], when the presence of hydrophilic carboxylate residues that bind cisplatin competed with the intermolecular hydrogen bonds.

## 3. Discussion and Conclusions

Studies on dendritic materials are based on the dendritic effect caused by their hyperbranched and hyperfunctional features [29]. In line with this concept, we present the synthesis of three systems selectively dendronized via methodologies that allow their final properties to be tailored for a specific application.

Thus, in system I, the dendronization process was applied directly on the surface of microbeads using the dendron, but the number of branches per unit of dendritic molecule was varied. The dendronized microbeads were studied as catalysts against the decomposition reaction of peroxide in aqueous medium. Catalysis in these systems was potentiated when a multivalent effect was present, since when dendrons with a higher number of branches were used, even at lower concentrations of surface modification (system Ch-BDGE-BBh), the microbeads exhibited the highest efficiency as catalysts, indicating that the multivalent effect is due to the proximity and cooperative effect of the periphery groups. This type of interaction has many advantages over those of a monovalent nature since the binding can be collectively much stronger than the corresponding sum of monovalent interactions. The synergistic effect [30] of the multivalent interactions therefore renders this system as a potentially successful catalyst with high selectivity.

**Figure 12:**
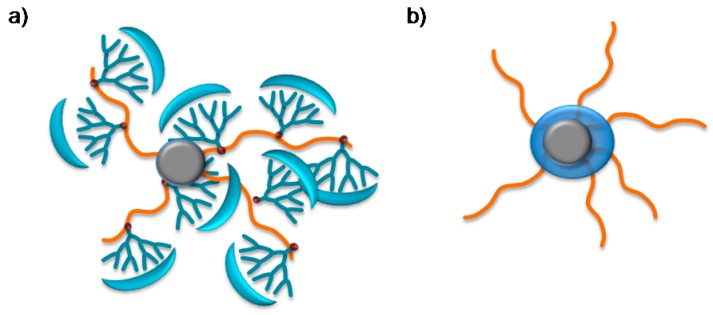
(**a**) Dendronized umbrella effect; (**b**) Core-brush effect.

In the case of system II, nanostructured surfaces of PP films, the Behera´s amine dendron was used as the monomer to obtain dendronized NPs-g-PABA, which were later used to modify the surface of PP. In previous studies performed in similar systems, PP surfaces were modified with TiO_2_ NPs without chemical modification (PP-NPs). However, in these cases, surfaces with superhydrophobic properties were only obtained using the highest NP concentrations and the HCA were always higher than 10, making it impossible to obtain self-cleaning properties. The use of a dendritic monomer allowed us to drive the silane/xylene ratio to obtain superhydrophobic or superhydrophobic and self-cleaning properties. This can be explained by the fact that the use of a dendritic monomer gives rise to a greater surface shielding of the NPs with lower concentrations of monomers, obviating the need for long chains of polymers to achieve this. We postulate that in these cases, an “umbrella” architecture is observed in the NPs rather than a core-brush (Figure 12) and for this reason, the properties of superhydrophobicity are achieved at a low monomer/mass NP ratio.

In system III, corresponding to smart NGs, the dendritic monomer played a preponderant role in the management of the hydrogen bonding interactions as a function of the pH of the medium and in the hydrophobic/hydrophilic balance that determined the value of Tcp. The results obtained with NiPAm-co-dendron 5% systems therefore enabled us to affirm that the presence of three carboxylic residues in close proximity in the dendritic monomer could explain the control/release behavior of the system at pH 5. In this specific condition, NiPAm-co-dendron 5% presented a rapid release of cisplatinum under endolysomal conditions, making the system a potential nanocarrier with interesting control/release properties for cancer therapy.

In conclusion, with the described systems we were able to achieve final properties for specific potential applications by using different synthetic methodologies for the dendronization process, allowing for optimal spatial arrangements of the dendron. These arrangements introduced a dendritic effect that acted synergistically on the structure/property relationship of the different systems. Another important point is that in all cases, the properties discussed for each system were achieved at low dendron concentration, which further justifies the use and advantages of dendronization processes.

## Figures and Tables

**Table 1:** Comparative properties between PP surfaces nanostructured with TiO_2_ NPs with and without chemical modification by dendronization.

Properties	PP-NPs		PP-(NPs-g-PABA) Silane/Xylene Ratio	
			5/95	10/90
[NPs] (mg/mL in xylene)	1.5	15	10	10
CA	141	160	158	162
HCA	14	21	46	8
RMS (µm)	1.7	1.1	1.2	1.0

**Table 2:** Cloud point temperature (Tcp) and diameters in aqueous solutions measured by dynamic light scattering of NiPAm-based nanogel. Adapted from G.N. Rimondino et al. [25].

NG	Tcp (°C)			Size ^a^ (nm)	
	pH: 3	pH: 5	pH: 7.4	T = 15 °C, pH 3	T = 45 °C, pH 3
NiPAm 100%	33.8	33.8	33.2	146 (0.108)	>1000 (0.379)
NiPAm-co-ABC 2%	30.4	36.2	>65	103 (0.138)	>1000 (0.369)
NiPAm-co-ABC 5%	27.0	35.5	>65	92 (0.136)	>1000 (0.454)
NiPAm-co-ABC 10%	21.4	31.8	>65	200 (0.210)	>1000 (0.274)

^a^ Values in parentheses indicate the polydispersity index (PDI) of each sample.
